# Case Report: Twisted subbasal corneal nerve fibers in a case of neurofibromatosis type 1

**DOI:** 10.3389/fnins.2026.1874737

**Published:** 2026-08-10

**Authors:** Yu Zhao, Qi Ren, Pengxia Wan

**Affiliations:** Department of Ophthalmology, The First Affiliated Hospital, Sun Yat-sen University, Guangzhou, China

**Keywords:** corneal nerve fiber, *in vivo* confocal microscopy, neurofibromatosis type 1, ocular surface, subbasal nerve plexus

## Abstract

**Background:**

Previous studies of neurofibromatosis-related neuropathy have primarily focused on large-fiber neuropathy. We report twisted subbasal corneal nerve fibers as a descriptive corneal nerve abnormality associated with neurofibromatosis type 1 (NF1), thereby enhancing the understanding of ocular manifestations of neurofibromatosis.

**Case presentation:**

A 24-year-old female patient with a history of NF1 presented with dry eye symptoms and a left orbital lesion detected on computed tomography. Slit-lamp examination showed punctate epithelial keratitis in both eyes and a ring-shaped superficial stromal scar in the right eye. Magnetic resonance imaging demonstrated lesions compatible with NF1-associated neuroglial involvement, including a left orbital/optic pathway lesion. IVCM revealed thickened and tangled subbasal corneal nerve fibers with a reticular appearance in the right eye and focal thickening and twisting of subbasal nerve branches in the left eye. Quantitative analysis showed that corneal nerve fiber density was reduced to 6.25 n/mm^2^ in both eyes. The right eye had no detectable nerve branches (CNBD = 0), whereas the left eye had a branch density of 31.25 n/mm^2^.

**Conclusion:**

This case describes qualitative and quantitative subbasal corneal nerve abnormalities in a patient with NF1. These findings support the hypothesis that corneal nerve alterations can occur in NF1.

## 1 Background

Neuro-oculocutaneous syndromes are characterized by systemic hamartomas involving the central and peripheral nervous systems, eyes, skin, and other organs. Neurofibromatosis type 1 (NF1) is a common neuro-oculocutaneous syndrome characterized by multiple melanocytic lesions, including *café-au-lait* macules, as well as neuroglial lesions such as plexiform neurofibromas and optic pathway gliomas (Kresak and Walsh, 2016). Ocular involvement is frequent and can contribute to diagnosis and disease monitoring (Fisher et al., 2012). Historically, the ophthalmic evaluation of NF1 has focused primarily on Lisch nodules detected by slit-lamp examination and optic pathway gliomas identified by magnetic resonance (MR) imaging. Evidence of small-fiber neuropathy has been reported in NF1 (Barnett et al., 2019), but its ocular surface manifestations remain incompletely understood. Corneal nerves are accessible to non-invasive imaging with *in vivo* confocal microscopy (IVCM), and changes in the subbasal corneal nerve plexus have been investigated in ocular surface disease, infectious keratitis, corneal surgery, diabetes, and other systemic neuropathic disorders (Cruzat et al., 2010; Gad et al., 2022). In NF1, previous ocular surface studies have suggested reduced tear stability and decreased corneal sensitivity, raising the possibility that corneal nerve alterations may be part of the disease spectrum (Moramarco et al., 2020). However, qualitative IVCM findings alone cannot establish the presence of small-fiber neuropathy. In this study, we describe a case of NF1 with twisted and irregular subbasal corneal nerve fibers observed by IVCM and discuss these findings as descriptive corneal nerve abnormalities that require further quantitative and functional confirmation.

## 2 Case presentation

A 24-year-old female patient with a history of NF1 was referred from a local hospital after computed tomography (CT) identified a left orbital mass. She reported dry eye symptoms for approximately 2 weeks but had no obvious change in vision, pain with eye movement, headache, dizziness, or tinnitus. She had previously undergone surgery for an acoustic neuroma. The diagnosis of NF1 was based on previous clinical documentation and typical cutaneous findings.

**Figure 1:**
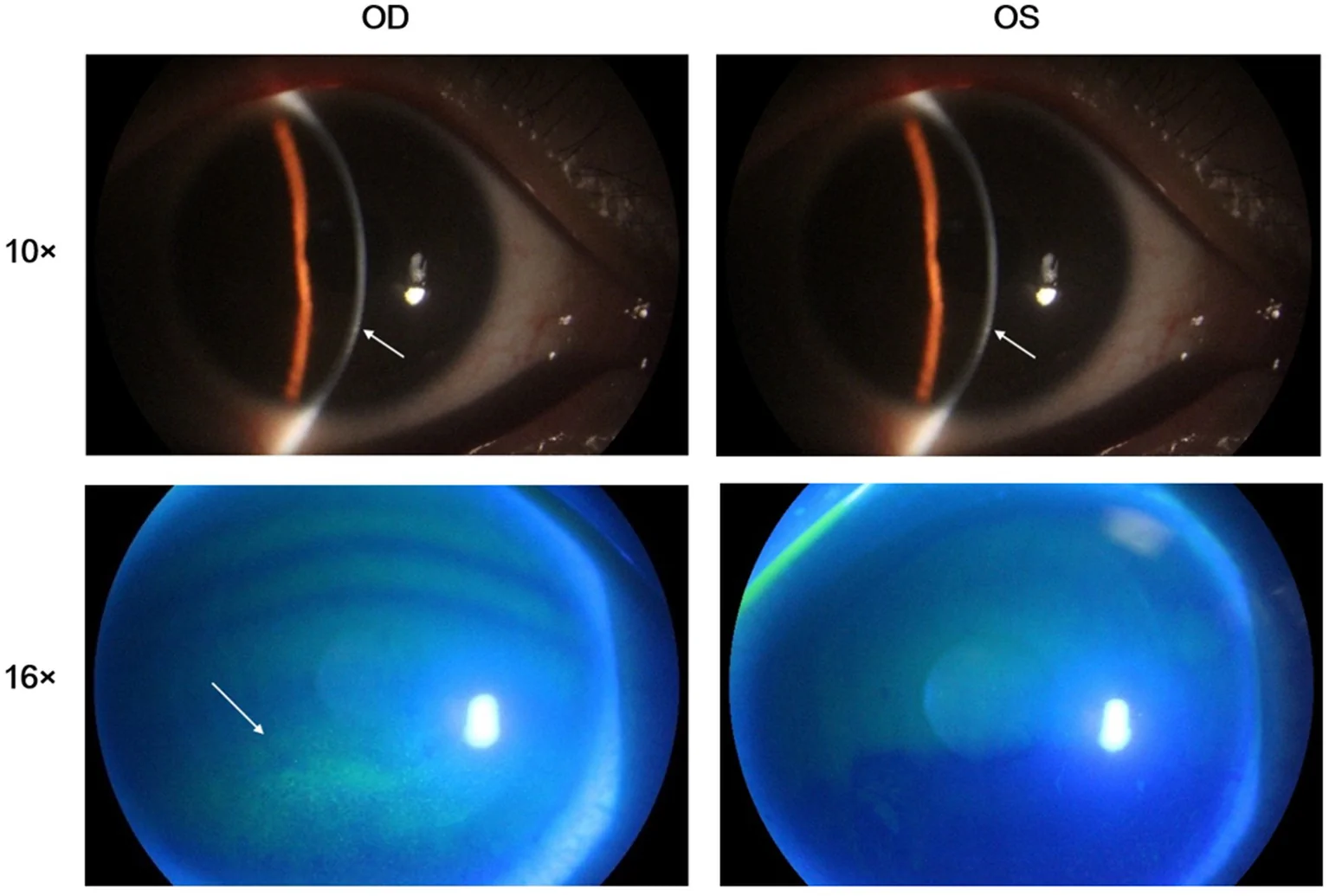
Punctate epithelial keratitis in both eyes. The right eye showed a ring-shaped superficial stromal scar and scattered punctate epithelial damage in the palpebral fissure area with positive fluorescein staining. The left eye showed punctate epithelial damage.

On presentation, the skin showed multiple uniformly hyperpigmented macules of varying size. Best-corrected visual acuity (BCVA) was 0.5 in the right eye (OD) and 0.7 in the left eye (OS). Hertel exophthalmometry measured 18 mm OD and 18 mm OS at a base of 112 mm, which was within the normal range (<22 mm). On slit-lamp examination (Figure 1), the right eye showed a ring-shaped scar in the superficial corneal stroma and scattered punctate epithelial damage in the palpebral fissure area with positive fluorescein staining. The left eye also showed punctate epithelial damage. The remaining anterior segment examination was unremarkable in both eyes. Ocular alignment and ocular motility were normal.

**Figure 2:**
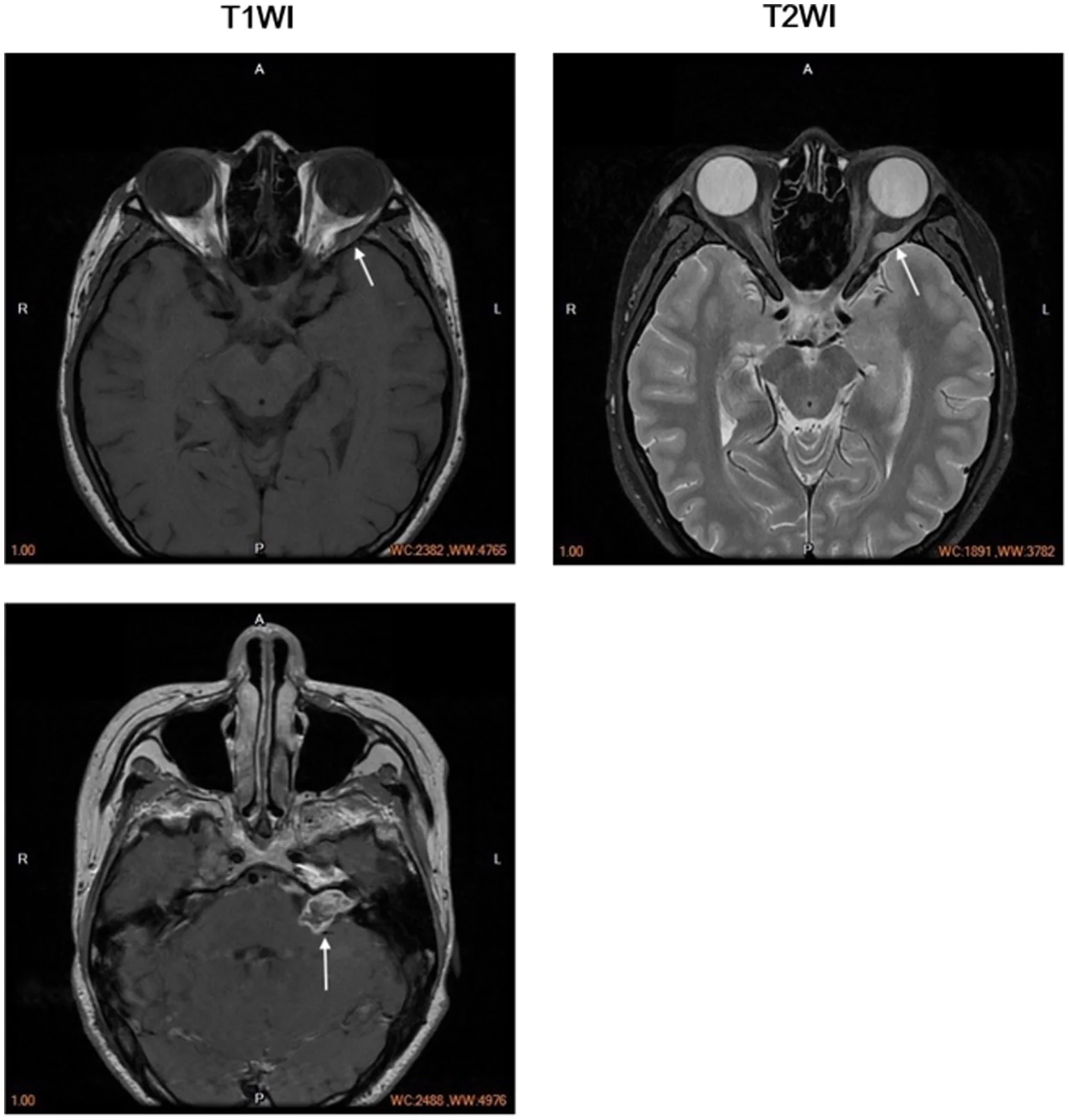
MR images. A nodule that appeared isointense on T1-weighted imaging and hyperintense on T2-weighted imaging was observed superior to the lateral rectus muscle of the left eye. The lesion measured approximately 6 mm x 8 mm, had a clear margin, and showed marked homogeneous enhancement. Additional lesions were observed along the left auditory nerve, bilateral trigeminal nerves, cervical spinal cord, subdural region of the cervical spinal canal, and intracranial meninges, with heterogeneous enhancement and patchy non-enhancing areas.

MR imaging (Figure 2) showed an isointense nodule on T1-weighted imaging and a hyperintense nodule on T2-weighted imaging superior to the lateral rectus muscle of the left eye. The nodule measured approximately 6 mm × 8 mm, had a clear margin, and showed marked homogeneous enhancement. Multiple T1-isointense and T2-hyperintense nodules or masses were also identified along the left auditory nerve, bilateral trigeminal nerves, cervical spinal cord, subdural region of the cervical spinal canal, and intracranial meninges. These lesions showed heterogeneous enhancement, with patchy non-enhancing areas suggestive of necrosis. Overall, the imaging findings were considered compatible with space-occupying lesions associated with NF1.

No diplopia was noted on clinical motility testing, and visual field testing showed no significant abnormalities in either eye. The final clinical diagnosis was neurofibromatosis with a left orbital NF1-associated lesion and punctate epithelial keratitis in both eyes.

## 3 IVCM

### 3.1 IVCM acquisition

**Figure 3:**
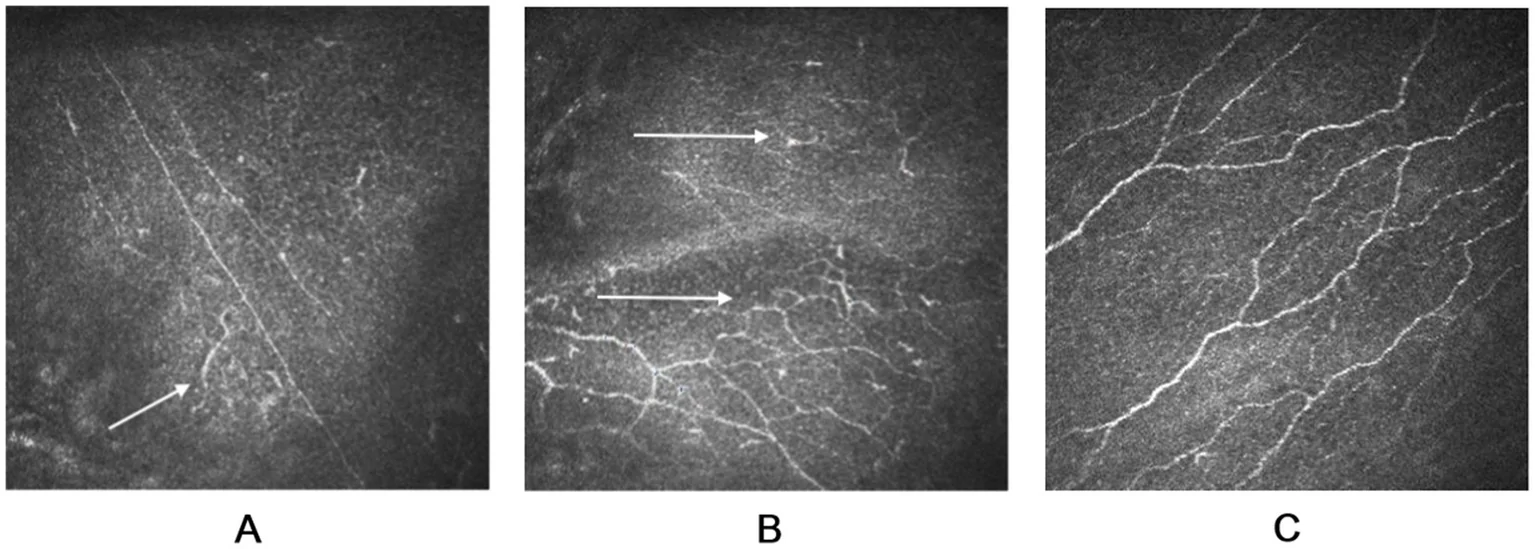
Subbasal nerve plexus images of the central cornea acquired by IVCM (HRT 3/RCM; 63×; 400 × 400 μm). **(A)** Right eye of the patient. White arrows point to thickened and tangled fibers forming a reticular pattern. **(B)** Left eye of the patient. White arrows indicate focal thickening and twisting of nerve branches. **(C)** Healthy control (26-year-old female patient) imaged under the same protocol for comparison, demonstrating regularly oriented, thin, non-tortuous nerve fibers with normal branching. The healthy control image **(C)** is included for illustrative purposes to show the typical appearance of the subbasal nerve plexus under the same imaging protocol.

IVCM was performed using the Heidelberg Retina Tomograph 3 with Rostock Cornea Module (HRT 3/RCM; Heidelberg Engineering, Heidelberg, Germany), with 63 × magnification and a 400 × 400 μm field of view. Imaging was performed after the application of topical anesthesia and coupling gel. The central cornea and the clinically apparent lesion areas under slit-lamp examination in both eyes were scanned. Images with optimal sharpness and focus, minimal motion artifacts, and representative visualization of the subbasal nerve plexus were selected for presentation. For each eye, one representative image of the central cornea with optimal visualization of the subbasal nerve plexus was selected for quantitative analysis. A representative image from an age-matched healthy female (26 years old) participant imaged under the same protocol was included for illustrative purposes only and was not intended as a formal comparator (Figure 3C). Written informed consent for publication of clinical images was obtained from both the patient and the healthy control participant.

### 3.2 Quantitative analysis

Quantitative analysis of corneal nerve fiber density (CNFD), branch density (CNBD), fiber length (CNFL), total branch density (CTBD), fiber area (CNFA), fiber width (CNFW), and fractal dimension (CFracDim) was performed using ACCMetrics (University of Manchester, UK), a plugin for ImageJ (NIH, United States). The central cornea (400 × 400 μm) was analyzed for each eye. For each eye, multiple scans of the central cornea were acquired, and one representative image (400 × 400 μm) with optimal focus, minimal motion artifacts, and absence of pressure lines was selected by two independent examiners for quantitative analysis. Therefore, the reported parameters represent measurements from a single representative image per eye rather than averaged measurements across multiple images. The same acquisition protocol was used for both eyes to ensure consistency.

### 3.3 Qualitative findings

In the right eye, the subbasal corneal nerve fibers appeared thickened and tangled, forming a reticular pattern, with a small number of dendritiform/Langerhans cells. In the left eye, some branches of the subbasal corneal nerve fibers were thickened and twisted. Although the fibers appeared morphologically thickened and tangled on qualitative assessment, quantitative CNFD was markedly reduced (Table 1). Stromal cells in both eyes appeared quiescent (Figure 3). The endothelial cell density was 2,431 ± 35 cells/mm^2^ in the right eye and 2,916 ± 45 cells/mm^2^ in the left eye.

**Table 1:** Quantitative corneal nerve parameters in both eyes.

Parameter (unit)	Right eye (OD)	Left eye (OS)
CNFD (n/mm^2^)	6.25	6.25
CNBD (n/mm^2^)	0	31.25
CNFL (mm/mm^2^)	5.73	12.02
CTBD (n/mm^2^)	6.25	93.74
CNFA (mm^2^/mm^2^)	0.0037	0.0086
CNFW (mm/mm^2^)	0.0207	0.0235
CFracDim	1.3828	1.4342

Quantitative results are summarized in Table 1. CNFD was 6.25 n/mm^2^ in both eyes. The right eye had a CNBD of 0 n/mm^2^ and a CTBD of 6.25 n/mm^2^; the left eye had a CNBD of 31.25 n/mm^2^ and a CTBD of 93.74 n/mm^2^. CNFL was 5.73 mm/mm^2^ in the right eye and 12.02 mm/mm^2^ in the left eye. Fractal dimension was 1.38 in the right eye and 1.43 in the left eye. These values are substantially lower than the 5th percentile normative values for healthy female participants aged 16–25 years (CNFD: 20.07 n/mm^2^, CNBD: 34.01 n/mm^2^, and CNFL: 15.08 mm/mm^2^) reported by Tavakoli et al. (2015). Similar corneal nerve changes have been reported in patients with acoustic neuroma (Chen et al., 2025).

## 4 Discussion

Several ocular manifestations have been described in patients with NF1, including Lisch nodules (Richetta et al., 2004; Antoniolli et al., 2021), optic pathway gliomas, orbital and eyelid neurofibromas, congenital glaucoma, choroidal abnormalities (Viola et al., 2012), and retinal microvascular alterations (Parrozzani et al., 2018). These findings are primarily pigmented, neuroglial, vascular, or structural manifestations. By contrast, the present case focuses on microscopic changes in the subbasal corneal nerve plexus, which are not visible on routine slit-lamp examination and require IVCM for visualization.

Recent studies have suggested that the ocular surface may also be involved in NF1. Moramarco et al. reported reduced tear stability and decreased corneal sensitivity in patients with NF1, supporting the possibility that corneal nerve involvement may occur in this disease (Moramarco et al., 2020). Barnett et al. reported evidence of small-fiber neuropathy in NF1 using systemic neurologic assessment (Barnett et al., 2019). Our case provides *in vivo* quantitative corneal nerve findings that are consistent with these observations. The patient had dry eye symptoms and punctate epithelial damage, and IVCM demonstrated bilaterally reduced nerve fiber density (6.25 n/mm^2^) with asymmetric branching: the right eye had no detectable branches, whereas the left eye retained a branch density of 31.25 n/mm^2^.

It should be noted that corneal nerve tortuosity and irregular morphology are not specific to NF1 or small-fiber neuropathy. IVCM has demonstrated subbasal nerve changes in several ocular and systemic conditions, including dry eye disease (Kheirkhah et al., 2015). Furthermore, the patient’s dry eye disease, punctate epithelial keratitis, and corneal scar may independently contribute to the observed corneal nerve morphology. It is also worth noting that similar tortuous and thickened nerve fibers can be observed in other conditions, such as post-herpetic keratopathy and diabetic neuropathy, indicating that this morphology is not pathognomonic for NF1. Therefore, similar-appearing parallel or tortuous nerve fibers may reflect multiple mechanisms, including inflammation, epithelial compromise, nerve degeneration, and attempted regeneration. The absence of nerve branches in the right eye could be due to the local corneal scar, asymmetric disease, or sampling bias.

In conclusion, we describe qualitative and quantitative IVCM findings of thickened and tangled subbasal corneal nerve fibers in a patient with NF1. These findings expand the descriptive spectrum of ocular surface changes that may be observed in NF1, but they do not by themselves establish small-fiber neuropathy. Larger studies with standardized imaging, quantitative nerve metrics, corneal sensitivity testing, healthy controls, and systemic neurologic evaluation are needed before the frequency or diagnostic significance of corneal nerve abnormalities in NF1 can be determined.

## Data Availability

The original contributions presented in the study are included in the article/supplementary material, further inquiries can be directed to the corresponding author.
